# Entrepreneurship and geography—some thoughts about a complex relationship

**DOI:** 10.1007/s00168-021-01091-w

**Published:** 2021-12-03

**Authors:** Rolf Sternberg

**Affiliations:** Institute of Economic and Cultural Geography, Leibniz University Hannover, Schneiderberg 50, 30167 Hannover, Germany

**Keywords:** R19, L26, R11

## Abstract

This review article sheds a light on the complex and hitherto under-researched relationship between geography and entrepreneurship. This relationship is considered to be interdependent. Both directions are discussed. The paper also describes the perspectives of both academic disciplines involved in regional entrepreneurship research, namely (geographically sensitive) economics and management studies on the one hand, and economic geography on the other. Based on a comprehensive overview of the theoretical and empirical literature on regional entrepreneurship, several research gaps are identified that could be helpful for designing future research. Some have strong relevance for government policy, which has recently paid much more attention to entrepreneurship than in the past (e.g. related to the entrepreneurial ecosystem approach), but which rather rarely has been considered in academic evaluations so far. This paper ends with a suggestion for an agenda for future regional entrepreneurship research. Digital transformation with its potential for a disruptive transformation of economies and societies will provide an excellent and, of course, a currently not well-understood research field for regional entrepreneurship research.

## Introduction

Entrepreneurship and economic geography are both socio-economic phenomena that recently have grown significantly in terms of academic and policy relevance. Government policies to strengthen entrepreneurial activities are nowadays present in all industrialised countries and in most of the developing countries. They are considered an important means of supporting the economic development at the supranational level group of countries (like the EU), at the national level of selected countries, and at the sub-national level of regions (like the federal states within Germany or the USA), or even local communities. While surely not every territory has the potential to become the next Silicon Valley, which emerged from a very unique mixture of entrepreneurial spirit, start-up dynamics, and historical incidences, many policy-makers indeed do believe that entrepreneurial activities in the form of new ventures led by owner–managers have the potential to spur economic development. Partially due to these expectations among policy-makers and partially because research is emerging independently, we can observe an extreme increase in scientific publications, academic conferences, and journals on the subject over the last three decades. Entrepreneurship research has long left the narrow disciplinary boundaries that characterised the entrepreneurship field some decades ago. Presently, entrepreneurship topics have grown in popularity (while still inhabiting a small niche in the identified disciplines) in several more or less neighbouring academic disciplines like psychology, sociology, and economic geography (Obschonka and Stuetzer 2017; Bögenhold et al. 2014; Mayer and Leick 2019). This might be interpreted as a clear indication of the interdisciplinary nature of entrepreneurship itself, but that does not automatically mean that the identified academic fields always have close relationships and seek intense cooperations across disciplines when entrepreneurship research is conducted. Despite many valuable examples of such cooperations in recent years, real interdisciplinarity is still, as in other academic fields, more often an assertion than a practice.

It is worth mentioning that the understanding of entrepreneurship in general and of its relationship with other fields differs among entrepreneurship scholars of different academic disciplines. Let me just mention three of the most influential. For economic geographer Maryann Feldman (2001), “entrepreneurship is primarily a *regional* event” that is largely influenced by geography (Malecki 2009), albeit the original idea of “the entrepreneurial event” was introduced by management scholar Albert Shapero (1984). According to economist Erik Stam (2010, 141) “entrepreneurship is the result of the interaction between individual attributes and the surrounding environments”. Finally, leading management and entrepreneurship scholar Per Davidsson (2016, 62f) observes, “entrepreneurship research encompasses the study of processes of […] emergence of new economic ventures, across organizational contexts. This entails the study of new venture ideas and their contextual fit; of actors and their behaviors […], and of how the characteristics of ideas, actors and behaviors link to antecedents and outcomes on different levels of analysis”.

This paper focuses on the relationship between two of these academic fields, entrepreneurship and economic geography, and between both research objects with the same name. Scholars of several different academic fields contribute to entrepreneurship research, indicating that entrepreneurship is a “multi-level phenomenon” (Davidsson 2016, 21). This paper mainly concentrates on the two identified fields and consequently addresses the content overlap between them, i.e. the geographical aspects of entrepreneurship and the entrepreneurial aspects of economic geography, respectively.

Whenever entrepreneurship is considered from an academic perspective, it is necessary to define what is meant by this not self-explanatory term. In this paper, entrepreneurship is related to any kind of new businesses, as the term has been used in the Global Entrepreneurship Monitor (GEM) project for two decades. Following Sternberg and Wennekers (2005), entrepreneurship refers, first, to owning and managing a business on one’s own account and at one’s own risk and, second, to “entrepreneurial behaviour” in the sense of seizing an economic opportunity. For this paper, I define entrepreneurship as a combination of some elements of behavioural entrepreneurship with some aspects of the dynamic perspective of occupational entrepreneurship, therefore characterising new venture creation as the hallmark of entrepreneurship (Cooper 2003).

The paper is structured as follows. Following the introduction, the second section examines the relationship between entrepreneurship and economic geography from two perspectives. First, the role of geography within entrepreneurship is addressed, followed by the opposite perception, i.e. the role of entrepreneurship within economic geography. In the third section, a review of the current state of the art of geographically sensitive entrepreneurship research is given, divided into theoretical and empirical research. The fourth section is dedicated to the policy side of regional entrepreneurship research. The related opportunities and challenges are described. Based upon obvious research gaps, some suggestions regarding a regional entrepreneurship research agenda are proposed in the fifth and final section.

## Research concerning the relationship between (economic) geography and entrepreneurship

When elaborating on the relationship between economic geography and entrepreneurship, at least three perspectives can be distinguished: a theoretical, an empirical, and a disciplinary angle.

In terms of theory, a contextual turn in entrepreneurship research has obviously taken place during the last two decades (Zahra et al. 2014; Baker and Welter 2018). The context perspective may be interpreted as a reaction to the long-standing dominance of person-related entrepreneurship theory, which stressed the role of characteristics of the individual entrepreneur (or potential entrepreneur) to explain whether and why an individual becomes an entrepreneur or even a successful entrepreneur (for a kind of rollback, however, see Rauch and Frese 2007). Partially related to this contextual turn—and conceptionally a part of it—a spatial or geographical turn within economics has occurred as well in the last two decades. The geographical context of entrepreneurial activities may have an impact on the extent of individuals’ entrepreneurial activities, intentions, and perceptions in a given territory, and combined with personal characteristics of the named individuals, it may exert an additional effect on such activities, intentions, and perceptions. Related theories, like the knowledge spillover theory of entrepreneurship (Audretsch and Keilbach 2007, Mueller 2006), the entrepreneurial ecosystem approach (Alvedalen and Boschma 2017; Stam 2015; Wurth et al. 2021), or the explanation of the emergence of regional–sectoral clusters (Klepper 2007, 2009, 2010) more or less explicitly acknowledge that the geographical context plays a role at different (and interdependent) geographical levels ranging from the supranational to the national (as applied in the GEM project for two decades, www.gemconsortium.org) to the regional (i.e. the sub-national regions addressed in the REDI project, see Szerb et al. 2019), or even to the local level. Besides their role for explaining the entrepreneurial behaviour of individuals, several theoretical concepts explicitly acknowledge the geographical context as being relevant for conceptualising and measuring, from an aggregated perspective, entrepreneurial activities in regions or countries. The economic geography view on the theory of the firm (e.g. the resource-based view in the Penrosian sense (1959) applied in publications by Garnsey et al. 2006, Garnsey 1998, and Stam 2007) points to the relevance of firm-specific attributes and addresses the reasons for the growth of young firms in particular (Maskell 2001; Taylor and Asheim 2001). The concept of regional growth regimes, adapted from the much older concept of innovation regimes, stresses that the new (instead of the incumbent) firms are the drivers of regional economic development (Audretsch and Fritsch 2002). It argues that growth conditions differ between (sub-national) regions and that therefore the effects of entrepreneurial activities on regional growth also differ across regions. Thus, it might be useful to distinguish between different types of regional growth regimes because their framework conditions do significantly differ. The knowledge spillover theory of entrepreneurship (Audretsch and Keilbach 2007), while not explicitly referring to the geographical context, stresses that spillovers are distance-sensitive and thus more frequently occur within certain geographical boundaries rather than spreading worldwide. This precisely coincides with the numerous indications that new businesses are primarily founded—and are more successful—in the (local) region where the entrepreneur was living and working before he/she started the business (Dahl and Sorenson 2012). Finally, and more recently, the various attempts to conceptualise entrepreneurial ecosystems (EES) should be mentioned as it is considered by very different fields of entrepreneurship research (Alvedalen and Boschma 2017; Feldman et al. 2019; Brown and Mason 2017; Hayter et al. 2018; Mason and Brown 2014). While the emergence of this still quite new concept was driven by practitioners, often without a clear spatial perspective, it soon became obvious that EES show their strongest explanatory power at the sub-national, i.e. the regional, level (Malecki 2018b).

Second, there is a clear empirical perspective on the relationship between entrepreneurship and economic geography. In the past two decades, numerous empirical papers have tried to empirically assess the causes of entrepreneurial activities in certain geographical areas with an explicit consideration of regional context determinants. (See, for example, the Special Issues of “*Regional Studies*” 1984, 1994, 2004, and 2014 as well as review articles like those of Müller 2016 or Sternberg 2009.) This geographically sensitive stream of empirical entrepreneurship research applies increasingly sophisticated quantitative methods, but it still widely ignored qualitative ones or mixed method attempts that combine quantitative and qualitative techniques to collect and/or to use data (Stam 2010). One explanation for the increasing relevance of empirical research is the availability of several new sources of primary data that have emerged during the past 20 years such as those created by international consortia like GEM and REDI and global institutions such as the OECD and the World Bank. Also, the contribution of the start-up Genome project (most recently Gauthier 2020), beginning in 2015, should be mentioned here. As a consequence, the empirical gap in geographically sensitive entrepreneurship research has been reduced, but it still exists, in particular when it comes to interregional comparisons across countries or even continents. (See Bosma and Sternberg 2014 for an exception.)

Third, there is a disciplinary perspective on the identified relationship. Economic geography, as a sub-discipline of geography, and entrepreneurship are dedicated academic disciplines that are taught via many chairs at faculties, universities, and countries. Economic geography is an academic discipline that increasingly cares about entrepreneurial activities, but with (partially) specific theories and methods (different from those of non-geographic entrepreneurship scholars). In recent decades, despite significant overlapping between economics and economic geography regarding research objects and goals, the general relationship between both disciplines has been complicated. (See the debate about “lions and butterflies”, as described by Duranton and Rodríguez-Posé 2005, that became even stronger after the self-proclaimed economic geographer Paul Krugman received the Nobel Prize for Economics in 2008.) The relationship between the two disciplines was characterised as mutual ignorance despite the same research objectives (Sjöberg and Sjöholm 2002), evidenced, for example, in terms of very rare joint publications, despite considerable cross-citation (Sternberg 2015b). Some exceptions like those in the new field of evolutionary economic geography/economics and individual scholars from both fields confirm the named rule. Also, some scholars have broadened regional entrepreneurship research beyond evolutionary economic geography including Brekke (2015) and Roundy and Fayard (2019). In terms of entrepreneurship research, combined activities seem to me more frequent than in other research fields. Consider, for example, several joint publications of dedicated economic geographers like Maryann Feldman, Niels Bosma, and Rolf Sternberg together with economists like David Audretsch, Michael Fritsch, Zoltan Acs, and Michael Wyrwich.

### Role of geography in entrepreneurship research

When explaining entrepreneurial activities and perceptions of individuals and aggregates like cities, regions, or countries, contextual factors have recently gained significance, although they are clearly still not a major field of entrepreneurship research (Welter 2011; Davidsson 2016). One reason is that approaches concentrating on personal factors relating to the entrepreneur—that for long dominated entrepreneurship research—have relatively lost relevance because those factors only stood up to empirical analysis to a limited extent (Davidsson 2016). Personal factors alone cannot explain the entrepreneurship event, so context factors are becoming more popular. Nowadays, entrepreneurship is seen more often as “a generically social, a collective phenomenon” (Johannisson 2000, p. 306) that is influenced by contextual determinants. From a theoretical standpoint, the analysis of the contextual determinants of entrepreneurship, defined as the creation of organisations, is captured in the demand-side approach to entrepreneurship (Thornton 1999) mentioned in Fig. 3 of Sect. 3.1.

Geographical attributes belong to the contextual factors that may have an impact on entrepreneurial activities and the perceptions of individuals. Following Boschma's (2005) conceptualisation of proximities, other relevant context factors include the organisational, the social, the institutional, or the cultural context an individual is living in or has lived in the past. (See Sternberg, 2022 for the role of these proximities for entrepreneurship.) Geographical proximity is a contextual environment of a territory that has some—relatively easy to determine—boundaries (e.g. being located in a certain area). If a young or a nascent entrepreneur acts within these fields of impact (i.e. within such boundaries), he/she will be more or less influenced by such a context.

Several prominent entrepreneurship scholars stress the geography of entrepreneurial activity. Olaf Sorenson, the winner of the Global Award in 2018 for entrepreneurship research, argues that the geographical context, in a complex combination with other contextual factors and person-related determinants, affects entrepreneurial decision and behaviour—and the success of new ventures (Sorenson 2018, Dahl and Sorenson 2009, 2012; see also Rickne et al. 2018). In several publications, Stephen Klepper explains the genesis of regional–sectoral clusters of new industries to the geographically embedded spin-off processes, i.e. he acknowledges the important role of geographical attributes for distinct (and geography-specific) entrepreneurial processes (Klepper 2009; see also Agarwal and Braguinsky 2015). In the words of Davidsson (2016, 32): “For all its qualities as an entrepreneurship hotbed, Silicon Valley might not have turned out the right environment for launching the Ice Hotel”.

Another, more indirect indication for the relevance of geography in entrepreneurship research is that entrepreneurship (as well as regional economic development) is rather unevenly distributed across space, i.e. entrepreneurial activities, motivations, and perceptions differ more or less significantly between countries of a continent, between sub-national regions within a country, and between quarters of a city (Sternberg 2009). These kinds of spatial disparities are also observed for regional economic development—growth rates of economic indicators like GDP do also differ across countries, across sub-national regions within the same country, or between urban and rural areas within the same country (World Bank 2009). Furthermore, these two observations are interdependent: entrepreneurial activities do (to a degree) influence regional economic growth, while the latter also has an impact on the level of entrepreneurial activities in a given territory (Sternberg 2009). When one accepts that the explanation and description of economic growth of countries, regions, and cities belong to the core tasks of economic geography—and many economic geography scholars do (see, for example, the economic geography textbooks of Bathelt and Glückler (2018), Bröcker and Fritsch (2012) or the successful edited volumes like the New Oxford Handbook of Economic Geography (Clark et al. 2018)—the connection between entrepreneurial activities and economic geography is obvious.

### The role of entrepreneurship in economic geography research

Following this assessment, one might argue that regional economic development belongs to the core tasks of economic geography as an academic discipline. While not every economic geographer would support this assessment, the majority currently surely do. The logical next step would then be to elaborate on the importance of entrepreneurship for regional economic development. A decade ago, Rutten and Gelissen (2008, 1003) stated that “the importance of entrepreneurship for regional economic development is virtually uncontested in the (regional) economic literature”. Nowadays, such a statement would rarely be possible because numerous publications by economists and regional economists in particular and by (proper) economic geographers stress the role of entrepreneurship for regional development. Many regional economists like Fritsch (2013) have pointed to the role of entrepreneurship for regional development. In focussing on economic geographers, it is worth mentioning the work of Feldman (2014), Malecki (2018a), Baumgartner et al. (2013), Sternberg (2009), Scott (2006), and Bosma (2009).

Entrepreneurship in economic geography textbooks is more often explicitly considered than was the case in previous decades. If entrepreneurial activities are considered, they are often assessed with regard to economically very successful sub-national regions like the Silicon Valley, the Boston Route 128, or, more recently, Berlin in Germany. Former or current start-up hot spots (usually interpreted as spatial concentrations of entrepreneurial activities in certain cities or city regions) are in many cases assessed as being conducive to regional economic development. Following this short assessment, university curricular of economic geography is usually strongly connected to regional economic development or growth. This is understandable because the regional economic development of sub-national regions is among the most frequently taught topics of economic geography at universities.

In a nutshell, economic geographers seem to be less often engaged in entrepreneurship research than entrepreneurship researchers from economics/management studies are engaged in geographical issues, but there are many, mainly empirical, works. Economic geographers in particular have pointed to clear links between regional attributes and entrepreneurial behaviour and perceptions in the same regions. See, for example, the research of the strong economic geography group at Utrecht University, which includes entrepreneurship studies by Niels Bosma (2009), Erik Stam (2007), and Veronique Schutjens (Bosma and Schutjens 2011). There are also plenty of empirical studies based upon GEM data, which were used to empirically disentangle the role of entrepreneurship in regional economic growth and development. (For an overview, see Bergmann et al. 2014.) Economic geographers were and are still engaged in research about the entrepreneurship support infrastructure as a means to encourage and foster entrepreneurial activities and success. (See, e.g. Sternberg 2009 for an overview and Tamásy 2006 for the role of business incubators as one particularly popular instrument of local entrepreneurship support policies.) Also, and not that surprisingly, economic geographers quite often have shown strong empirical evidence that geographical context matters for entrepreneurship (Malecki 2012), e.g. in terms of local inertia and the seedbed hypothesis of the emergence of new ventures (Hayter 1997). In recent years, economic geographers have also shown that different spatial levels play a role in explaining the role of geography for entrepreneurship activities and have consequently applied multilevel techniques. (See, for example, Bosma 2009, Theodoraki and Messeghem 2017, or Hundt and Sternberg 2016.) The rather young evolutionary economic geography stream of literature argues that regional dynamics are mainly caused by new firms (and not by incumbent ones) located in these regions (Boschma and Martin 2010; Stam 2010). Finally, some economic geographers with a more relational perspective have enriched the rather recent debate about regional entrepreneurial ecosystems as they consider entrepreneurship as a social geographical phenomenon: these scholars are studying how different entrepreneurial environments develop and evolve within places and within an entrepreneurial ecosystem and how this influences a firm’s start-up strategy (Spigel 2018; Spigel and Harrison 2018). By considering social relationships as crucial for entrepreneurial activities, too, networks in all their facets are considered very important as well, as Malecki (2018a) has recently stressed.

## State of the art of geographically sensitive entrepreneurship research

**Fig. 1:**
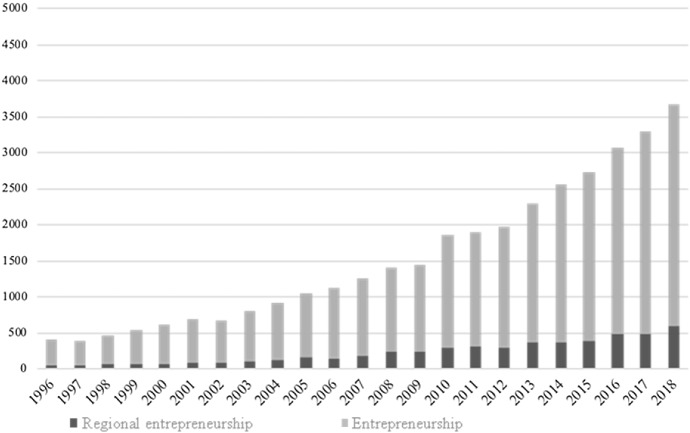
Number of publications on entrepreneurship or regional entrepreneurship: absolute growth 1996–2018

In general, there is an obvious increase in research activities in entrepreneurship in general and the geographical context in particular, measured by the absolute and relative frequencies of academic journal publications. (see Sternberg 2022 and Torre 2014.) Investigating Elsevier's Scopus databank, which covers about 23,500 peer-reviewed scientific journals (https://www.elsevier.com/__data/assets/pdf_file/0017/114533/Scopus_GlobalResearch_Factsheet2019_FINAL_WEB.pdf), reveals a clear absolute increase in the number of journal papers relating to entrepreneurship in general over the last two decades (Fig. 1). This analysis is restricted to the top 20 journals of three academic disciplines (geography, management, and economics) according to the Web of Science categorisation of academic fields and the 2018 Impact Factors. (A list of these 60 journals is available upon request.) An absolute increase is also observable for those entrepreneurship papers that discuss regional entrepreneurship. The following search strings show how both groups of entrepreneurship articles are defined.

Search strings (“articles” and “reviews” only, reference years 1996–2018):

- *Entrepreneurship* TITLE-ABS-KEY (("entrepreneur*" OR "new firm*" OR "spin-off" OR "start-up" OR "incubator*" OR "science park*" OR "technology park" OR "self-employ*") AND ("business" OR "econom*")) AND (LIMIT-TO (DOCTYPE, "ar") OR LIMIT-TO (DOCTYPE, "re")).

- *Regional Entrepreneurship* TITLE-ABS-KEY (("entrepreneur*" OR "new firm*" OR "spin-off" OR "start-up" OR "incubator*" OR "science park*" OR "technology park" OR "self-employ*") AND ("region*" OR "spatial*") AND ("business" OR "econom*")) AND (LIMIT-TO (DOCTYPE, "ar") OR LIMIT-TO (DOCTYPE, "re")).

**Fig. 2:**
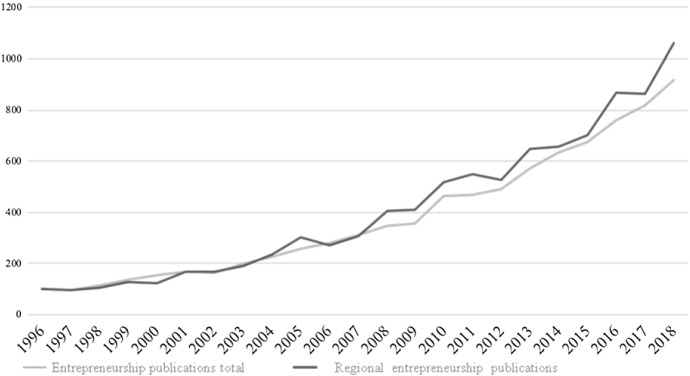
Number of publications on entrepreneurship or regional entrepreneurship: relative growth 1996–2018 (1996 = 100)

Comparing indices (1996 = 100) for both subgroups of articles, entrepreneurship in general and regional entrepreneurship, the latter group shows slightly higher increases over the period 1996–2018 (Fig. 2). Examples of review articles focussing on regional entrepreneurship, i.e. the role of geography for entrepreneurship, include Baumgartner et al. (2013), Trettin and Welter (2011), and Sternberg (2009).

### Theory

What is meanwhile widely accepted is that entrepreneurship is not under-theorised anymore, contrary to what Shane and Venkatamaran (2000) stated two decades ago. However, now “we may instead have a problem with overly strong and universal emphasis on theory” (Davidsson 2016, 64). Dissent exists about whether it is a problem for entrepreneurship research that these theories used in entrepreneurship are borrowed from other social science disciplines, while proper entrepreneurship theories in entrepreneurship research are rare. For the opposite positions, see Arend (2014), who observes a “continued atheoretical state of entrepreneurship research”, as well as Davidsson (2016, see also Parker 2018). Concerning the spatiality of entrepreneurship, according to evolutionary economic geographers (e.g. Stam 2010), various theories and concepts more or less explicitly address the spatial implications of entrepreneurship, for example, the inheritance of routines (Hodgson and Knudsen 2004), cognitive theories of innovation (Nooteboom 2000), and organisational ecology (Carroll and Hannan 2000). Such a spatial relevance also holds true for the so-called external enablers that Davidsson (2016, 235) described in his ideas to reconceptualise entrepreneurship opportunities. Such external enablers stand “for a distinct, external circumstance, which […] can play an essential role in eliciting and/or enabling a variety of venture development attempts by several entrepreneurial agents”. And they are, of course, geographically selective. Finally, it seems clear that emerging digital entrepreneurship is currently indeed still much under-theorised—and spatially blind (Kraus et al. 2019).

**Fig. 3:**
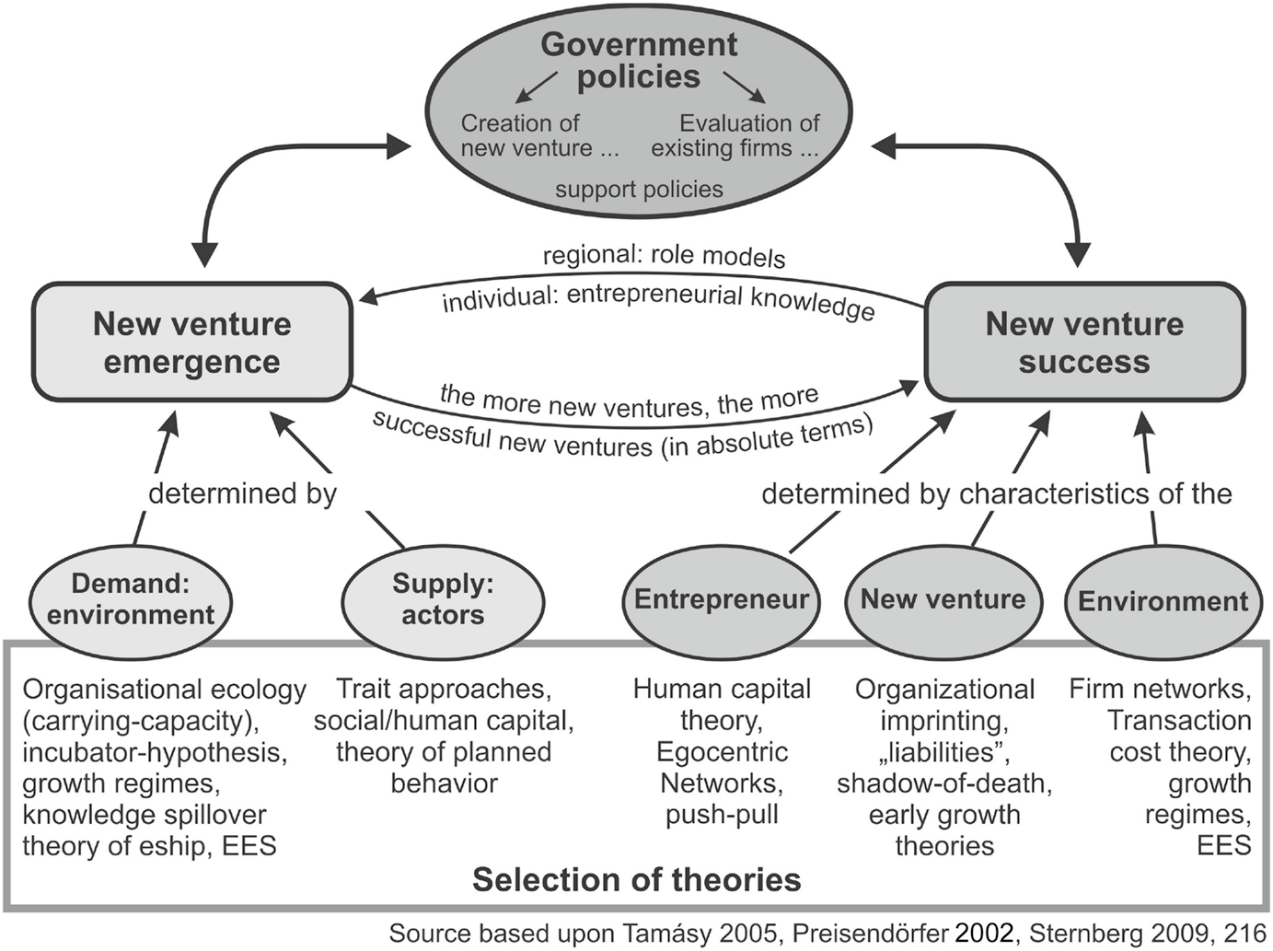
Entrepreneurship research: theories, topics, and policies

Figure 3 shows a rough overview of theories and concepts used in entrepreneurship research in the most recent two decades to explain new venture emergence and new venture success. Without claiming to be exhaustive, Fig. 3 covers those concepts that have gained a certain kind of reputation in entrepreneurship research across academic fields and that have rather often been used as a starting point for empirical studies.

**Fig. 4:**
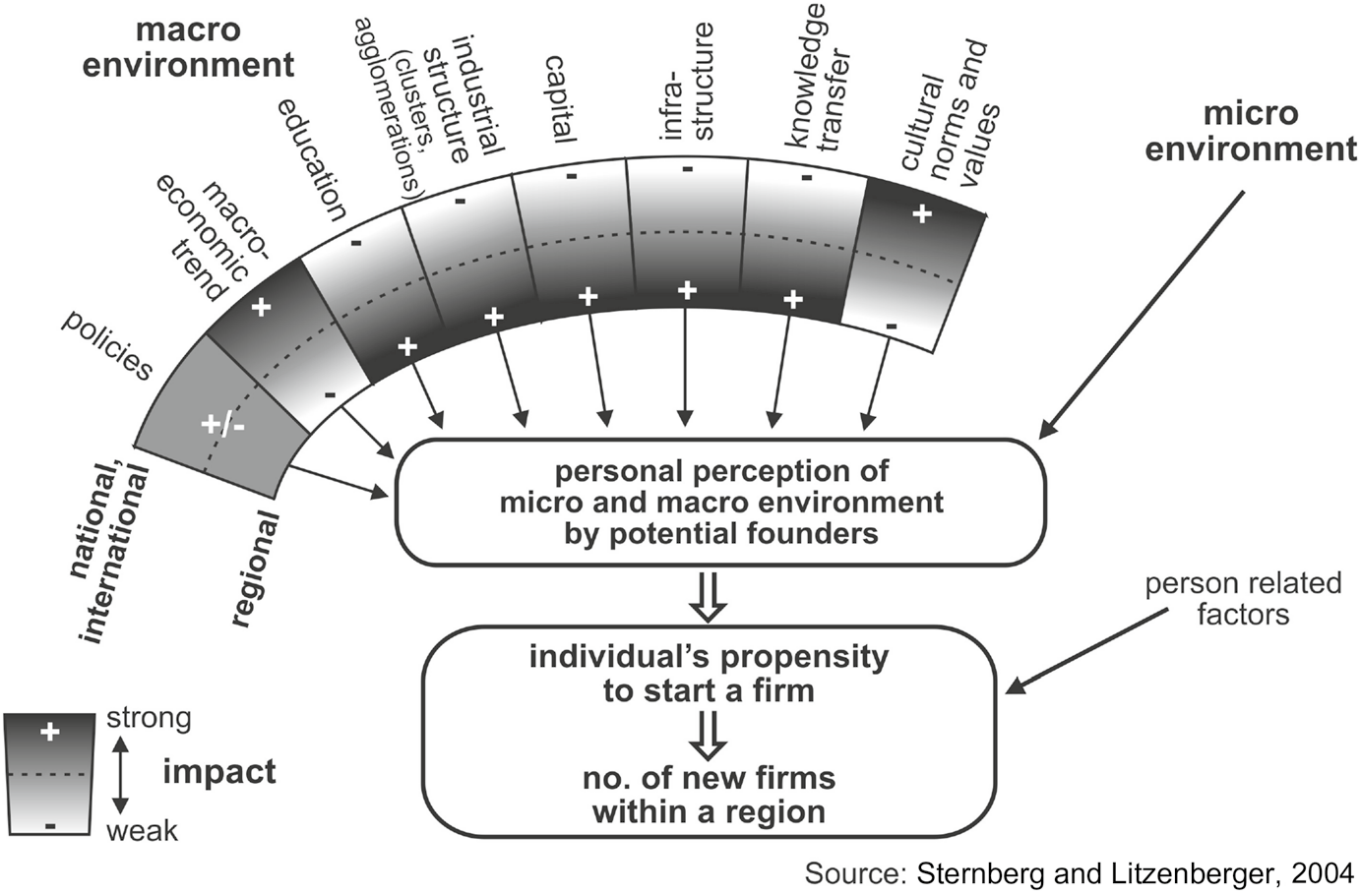
Spatial context’s impact on new venture emergence

As for the conceptualisation of the relationship between (economic) geography and entrepreneurship, much less progress has been achieved. The vast majority of regional entrepreneurship literature is empirical, not based on a widely accepted theoretical foundation or even a grand theory, but on a large number of very specialised theoretical assumptions for particular topics. (For an overview, see Sternberg 2015a.) Quite often, empirical papers on regional entrepreneurship are motivated by obvious empirical research gaps instead of theoretical gaps. Figure 4 shows an example of a multilevel perspective of the conceptualisation of regional entrepreneurship interpreting the geographical context at several levels: the macroeconomic level (e.g. attributes of the country or the sub-national region the entrepreneur or potential entrepreneur is living in), the micro-economic level (the social environment that includes friends, family, fools related to the entrepreneur, often characterised by a strong local focus), and the individual level (personal attributes of the entrepreneur him- or herself like trait factors, gender, age, and qualifications). These factors, embedded in a complex interrelationship shaped by geographical aspects, influence the individuals’ propensity to start (or not to start) a firm and, from an aggregate perspective, the absolute number of entrepreneurial activities in a given territory.

**Fig. 5:**
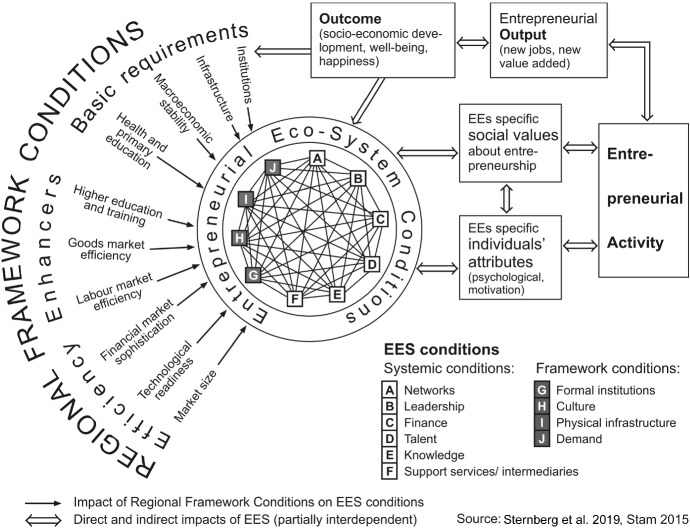
Conceptualisation of entrepreneurial ecosystems

In recent years, by far the hottest entrepreneurship topic has been entrepreneurial ecosystems (EES), as the significantly increased number of publications shows. Among the currently most popular attempts to conceptualise EES is the one first developed by Stam (2015). His interpretation and operationalisation based upon ten “elements” have the advantage of also being appropriate for empirically assessing the performance of an EES as seen through a geographical lens and both from a static and a dynamic perspective, as described in Fig. 5. (See also Sternberg et al. 2019a, b.) However, there are still many theoretical deficits (and even more empirical ones) regarding the EES concept (Alvedalen and Boschma 2017; Spigel 2018; Stam 2015; Wurth et al. 2021). The obvious danger is that history is repeating itself, similar to what happened with Michael Porter’s cluster concept in the 1990s or Richard Florida’s “Creative Class” concept a decade later, when still premature academic concepts were very quickly entering the policy arena and practitioners were developing policy instruments before independent scholars had created serious and comprehensive empirical evidence about the basic assumptions. (See also Sternberg 2012.)

### Empirics

Of course, it is not possible to describe the enormous amount of empirical research on regional entrepreneurship during the last two decades in a short section. (For overviews, see, e.g. Sternberg 2009, 2015a; Müller 2016, Fritsch 2013, see also Bouckenooghe et al. 2007.) I will nevertheless try to briefly summarise what seems to be relevant to establish a basis for the research agenda that will be presented in the last section.

First, the empirical (and interdependent) link between high regional (i.e. sub-national) levels of entrepreneurship and regional economic growth has been largely accepted and well proven (Fritsch 2013; Wagner and Sternberg 2004). Sectoral inertia (Armington and Acs 2002) and geographical inertia (Stuart and Sorenson 2003) are well-known aspects related to this link. Very well studied are the spatially different effects of entrepreneurship on the development of regional employment. (See, e.g. Fritsch and Schroeter 2011 and Fritsch and Wyrwich 2017.) The latter shows an obvious and positive relationship between a regional tradition or culture of self-employment and the effects of entrepreneurship on regional employment growth. However, the influence of the regional environment seems change as the entrepreneurship process progresses: it is lower for nascent entrepreneurs than for new entrepreneurs (for empirical evidence based on GUESSS data for student entrepreneurs see Bergmann et al. 2016) and it seems to be higher for start-up activities than for the later start-up growth and its survival. Factors explaining a firm’s birth are not the same as those explaining a firm’s growth. (See Stuart and Sorenson 2003; Sternberg 2009.) The surprisingly few empirical studies of regional survival rates show considerable differences across regions (Fritsch et al. 2006; Falck 2007; Acs et al. 2008), with much lower average survival rates in urban areas compared to rural ones. (For an exception, see Fotopoulos and Louri 2000.) The popular network theory of entrepreneurship (Brüderl and Preissendörfer 1998) shows rather inconsistent results on the relevance of regional networks for start-up success (Bloodgood et al. 1995; Shane and Stuart 2002; Lindelöf and Löfsten 2004; Davidsson and Honig 2003; Presutti et al. 2013; Sorenson 2018).

Second, localisation economies and, consequently, regional–sectoral clusters foster entrepreneurship (Rocha 2004) and, conversely, entrepreneurial activities do support the geographical concentration of economic activities (Feldman et al. 2005). Klepper’s (2009) spin-off model exploits knowledge from new firms’ parents, which provides a convincing explanation for the obvious spatial concentrations of new industries and entries in new markets, the results of which are clusters. Such clusters do show positive impacts on entrepreneurship, while pure geographical proximity alone (i.e. the co-location of firms in the same industry) is not sufficient to support entrepreneurship (Rocha and Sternberg 2005). Related to this—most clusters are more or less located in urban agglomerations—and despite increasing evidence of rural entrepreneurship (see e.g. Müller and Korsgaard 2018), entrepreneurship is, and always primarily has been, an urban event (Bosma and Sternberg 2014; Glaeser et al. 2010). While the positive effects of urbanisation effects on entrepreneurship are widely accepted (Bosma et al. 2008; Armington and Acs 2002), it should not be ignored that those agglomeration economies, whether as localization or urbanisation effects, may also show some negative effects on entrepreneurship (Letaifa and Rabeau 2013). Boschma (2005) suggests that too much geographical proximity can also result in lock-ins with negative effects on a firm’s innovativeness if the young entrepreneur communicates for too long and too exclusively with a few other network partners from within the same sub-national region. Thus, the geographical proximity that is available in regional–sectoral clusters is neither a prerequisite nor a sufficient condition for reinforcing the processes of knowledge acquisition and exploitation by co-localized start-ups (Boschma 2005). Lock-in risks are lower if the cluster and the region as a whole have relevant connections to other regions and external clusters (Sternberg 2007).

Third, regional entrepreneurship is the result of a complex interplay between person-related factors like demographic attributes (individual level) and context attributes, including the spatial context (meso-/macro-level of local areas, sub-national regions, countries), that require multilevel empirical analyses (Davidsson 2016). Related multilevel research shows that the regional environment does play a role both for entrepreneurial activity and for entrepreneurial attitudes, but measured in quantitative terms, its influence is much lower than that of attributes of the national environment and of the individual (i.e. person-related factors). (See Falck 2007; Bosma 2009; Hundt and Sternberg 2016.) Entrepreneurial attitudes and perceptions within the region, based upon individuals’ assessments, such as the fear of failure, perception of start-up opportunities, or perception of one’s own entrepreneurial skills, play an important part in explaining entrepreneurial activities in a given region. More importantly, they differ from one region to another (Obschonka et al. 2013; Tamásy 2006). More recent attempts to combine psychological knowledge and entrepreneurship research seem to offer a promising opportunity for interdisciplinary research on regional entrepreneurship to explain the role of regionally embedded psychological traits for entrepreneurial activities in the same region (Obschonka and Stuetzer 2017). First, these personality attributes seem to differ significantly across sub-national regions in several countries (Obschonka et al. 2013). Second, knowing a local entrepreneur who serves as a role model both in a positive (if he/she is successful) and in a negative sense (if he/she failed or is perceived to have failed) seems to play an important role for the regionally sensitive perception of entrepreneurship and self-employment and, consequently, for the level of regional entrepreneurship (Wyrwich et al. 2019).

Fourth, a rather young stream of empirical entrepreneurship research takes a long-term view on entrepreneurial activities and shows that entrepreneurial culture as a deeply embedded, regional resource provides an important explanation of the geography of entrepreneurship, particular via role model effects (Fritsch and Wyrwich 2017, 2018; Fritsch et al. 2019). These more recent empirical studies on entrepreneurship over (a long) time and space show that despite many changes in the environment, the spatial distribution of entrepreneurial activities seems to be rather persistent over time (Fotopoulos 2014; Fritsch and Wyrwich 2017). However, whether this will remain true in the future when digitisation as a disruptive technology unfolds its entrepreneurial consequences is still open for debate. Early attempts to measure “digital entrepreneurship” across countries, as presented by Autio et al. (2018) and the recent special issue of *Research Policy* (see Nambisan et al. 2019), are valuable first steps in this direction, but much has to be done there in the future. (See the research agenda in Sect. 5.)

## Policy opportunities and challenges

Since the 1980s, entrepreneurship has been very high on the policy agenda, starting in most of the European countries, but soon reaching many Asian countries and sub-national regions all over the world (McCann and Ortega-Argilés 2016; Lundström and Stevenson 2005; Audretsch et al. 2007; Leitao and Baptista 2009). Cities and their local governments have significantly contributed to the recent euphoria regarding entrepreneurial ecosystems as a practitioner-driven concept of territorially based entrepreneurship (Stam 2015). Entrepreneurial scholars in general and regional entrepreneurship scholars in particular should more explicitly consider this when they are conducting empirical entrepreneurship research because policy implications have not yet been sufficiently addressed in most basic entrepreneurship research. However, excellent (i.e. effective, efficient, and relevant) government policies to enhance entrepreneurship’s quality and quantity require sophisticated and objective evaluations by independent researchers who are well accepted in the academic world, to be conducted ex ante as well as ex post of the implementation of related support instruments. Of course, such an academic assessment of policy instruments to support regional entrepreneurship has to be an open process that may result in the positive or negative evaluation of certain measures. However, I do not agree with Shane’s (2009) verdict that every entrepreneurship policy is a bad policy. The various policy implications of the current popularity of entrepreneurship among many policy-makers provide valuable research opportunities, but the challenges should not be overlooked.

First, current entrepreneurship hype among governments and policy-makers in general and the exuberant enthusiasm regarding entrepreneurial ecosystems (mainly interpreted as spatially limited constructs; Malecki 2018b) in particular provide good opportunities for regional entrepreneurship research. I will point out only a few of them. Most of the entrepreneurship support policies are dedicated to regional (i.e. sub-national) or even local territories. That means that the responsible government agency has a clear geographical space for which it is responsible, and it consequently tries to support entrepreneurship in that very limited geographical space (e.g. a ministry of economic affairs of the German federal Land of Bavaria is exclusively aiming to support entrepreneurship activities located or potentially located in Bavaria instead of in other German federal states). The interregional or intercity competition for potentially mobile new firms and their founders might theoretically create some problems (a zero-sum game for a country’s government perspective and others), but given the strong local inertia and geographical embeddedness of most entrepreneurs and their firms, this is rarely the case.

The identified strong connection between the regional/local environment and entrepreneurship and the fact that most government support policies have a clear regional focus (instead of a national one) have one important advantage: if successful, such policies support (new firms in) those territories that will later profit from this support when these start-ups grow, create employment, and pay significant taxes. Those regions that sow do often also reap, unlike territories that have never sown. This kind of endogenous regional development may help policy-makers convince others when they are fighting for budgets for entrepreneurship support policies. Scholars doing related entrepreneurship policy evaluations of such place-based entrepreneurship support measures may in the future achieve more positive results compared with the few that have been conducted in the past when place-based entrepreneurship policies were less common (e.g. see US Government Accountability Office 2012 for the USA, National Audit Office 2013 for the UK, or OECD 2007 for the UK as reported in Fotopoulos and Storey 2019). One obviously quite important determinant of regional entrepreneurship activities—entrepreneurial culture—will, however, hardly be affected by policy-makers (or only in the extreme long run). In those regions that are characterised by such a persistent positive entrepreneurial culture, the latter was shown to be a good policy measure against external economic shocks (Fritsch and Wyrwich 2017).

While I am convinced that the identified policy perspectives offer more opportunities for regional entrepreneurship research than challenges, the latter should not be underestimated. Just to name a few of them, first, the “funding jungle” of too many support instruments, badly coordinated among each other, within the same regional environment, but also across geographical scales (often complained about in Germany, see, Sternberg et al. 2019a, b) may lead to frustration among potential entrepreneurs. One-stop agencies in a rather small and manageable region may help here. Second, entrepreneurship support policies need time to produce reliable positive results. Policy-makers, however, often do not have enough time to wait, but must think and act in much shorter time periods. Also, hype (like that for entrepreneurial ecosystems, see Brown and Mawson 2019) may soon end, although the good policy attempts have not yet been able to be successful. Thus, one must “mind the gap” (Kiese 2008) and not bet on the regional entrepreneurship horse too early because of a lack of empirical evidence, but one should also not give up too early because each policy instrument needs time to be effective. Government policies in favour of entrepreneurial ecosystems, the currently most popular entrepreneurship concept with strong relationships to geographical environments, obviously did not mind the gap. Most of them were created very quickly and before solid empirical data about the specific region/city were available. Such related policies indeed lack a valid theoretical foundation, so it is still open for debate whether entrepreneurship is a cause of or a response to economic change. Such government policies run the risk of treating the symptom rather than the cause, as Fotopoulos and Storey (2019) have rightly argued. However, one has to admit that very recently more and more empirical studies on regional EES are published and this may help to reduce the cited gap in the near future. (See, e.g. Iacobucci and Perugini 2020 and several of the citations therein.) Third, an important challenge is related to the dynamics of entrepreneurship activities over time: spatial entrepreneurship patterns within a country seem to be rather persistent over time (Fritsch & Wyrwich 2014, 2017) despite three decades of entrepreneurship support policies. Although that aspect has often been ignored in the research, one may conclude that entrepreneurship support policies have been unable to significantly change these patterns.

Finally, I offer one last remark on entrepreneurial ecosystems. Most academic interpretations of this concept consider them not to be policy-driven, but to be entrepreneurial-driven. That is, local entrepreneurs in a more or less self-organised process contribute to the development of such a system. Government policy is only one, and by far not the most important, agent in such entrepreneurial ecosystems. My conclusion is that policy-makers should indeed be completely silent in the discussion dealing with potential entrepreneurial ecosystems that do not yet exist (or may never emerge). However, if entrepreneurial ecosystems do exist, government policy should at least be one agent in the game because a lack of self-control within an EES may lead to under-exploitation of entrepreneurial ecosystems’ potentials.

## Research gaps and proposals for a research agenda

The earlier sections can be interpreted as a clear indication that much has been achieved both regarding the role of geography in entrepreneurship research and regarding the role of entrepreneurship for economic geography. However, several old or new blind spots persist in the research on the relationship between geography and entrepreneurship. The most relevant of these research gaps are therefore briefly summarised in the following. Where it makes sense, I distinguish between the gaps at methodological level and the gaps regarding relevant topics.

Starting with a more general remark on topics, it is helpful to note that much progress has been achieved in spatially interested (and not spatially blind) empirical research during the last two decades, partially due to improved and/or new databases and surveys. However, a new lack of adequate theories has emerged in terms of digitisation and its impact on regional entrepreneurship. Also, much more empirical testing of the rather young entrepreneurial ecosystem concept is required before policy-makers can seriously trust it when they are designing entrepreneurship support policies. (See also Wurth et al. 2021.) If region types are considered, at least some scholars (Müller and Korsgaard 2018) argue that entrepreneurship could also be a rural event, not an exclusively urban one, as the majority of regional entrepreneurship publications might suggest (e.g. Bosma and Sternberg 2014). To theoretically and empirically check for the ongoing relevance of the agglomeration effect argument, more studies on rural entrepreneurship (or studies comparing rural and urban entrepreneurship) are required to explain the differences. Also, “urban” is quite a context-dependent attribute that might be defined rather differently between countries worldwide. Such a revised test of the urban entrepreneurship argument should also consider digitisation and its general opportunities to reduce the relevance of physical distances, which will also favour rural regions.

From a methodological and data-related research perspective, studies stressing the quantity instead of the quality of entrepreneurial activities were much more frequent in the past. Given the significantly different impact of both attributes for entrepreneurship effects on, e.g. regional economic development, this underrepresentation of the quality aspect should be considered a research gap. Guzman and Stern (2015) have made a creative attempt to study the quality of entrepreneurship in a region, but their method is hardly transferable to regions outside the Silicon Valley. As for methods there is definitely a need for more qualitative efforts in empirical regional entrepreneurship research. (See, e.g. the experience sampling method by Uy et al. 2010 or some ethnographic studies.) An increase in the number of qualitative studies will not necessarily happen at the expense of quantitative attempts, but in an often fruitful (but rarely exercised) combination of quantitative and qualitative empirics within the same study. Because culture is considered an important cause that is less often analysed, more direct measurement of entrepreneurship such as a regional entrepreneurship culture is needed (e.g. Stuetzer et al. 2016). Qualitative attempts may be helpful here. Research gaps refer to the relevant mechanisms for creating such a region-specific culture of the mechanism to transfer it across generations and to the respective channels.

Finally, it cannot be overlooked that in general (as for most entrepreneurship topics), there is a strong dominance of academic studies, authors, and contents related to Northern American and Western European countries and sub-national regions. It is more than plausible to argue that the observed regional entrepreneurship patterns in these areas are not representative of the rest of the world, so regional entrepreneurship research stemming from and dealing with entrepreneurship processes in the emerging economies and developing countries is currently an important research gap.

Basing this progress in many fields on the complex relationships between geography and entrepreneurship, but also considering the identified research gaps, the following topics should be part of a future research agenda. Several of these topics were already identified by Stam (2010) ten years ago, while others were not.

First, entrepreneurial ecosystems as the currently most popular concept that is primarily interpreted through a geographical and regional lens deserve much more empirical research (Wurth et al. 2021). To empirically cover the complex relationship within a regional entrepreneurial ecosystem between actors requires new data and new data analysis techniques (Credit et al. 2018; Nightingale and Coad 2014). Also, in additional to “top-down” approaches (which focus on the actors and factors that make up an ecosystem), more “bottom-up” approaches are needed that examine how entrepreneurs use their ecosystems to acquire the resources, knowledge, and support they need.

Second, the role of digitisation in the relationship between geography and entrepreneurship has the potential to become one core topic of future regional entrepreneurship research. This research may be motivated by conceptual and theoretical weaknesses, by (an understandable) lack of empirical evidence, or by the intention to develop policy instruments to deal with the consequences of digitisation on the identified relationship. In such research, digital entrepreneurship should not be considered aspatial or even footloose because there is spatiality of the digital infrastructure (which I consider the “hardware” component of digitisation) and of digital competences and skills of entrepreneurs, employees, and the population in a given region (the “software” component). If we accept this spatiality of the hardware component (fast Internet service is available at higher quality in urban areas than in rural ones) and the software component (large urban agglomerations, on average and measured by relative indicators, do employ more people with higher digital competence than rural areas), one conclusion is that neither digitisation nor the Internet will end up in the “death of distance” (Cairncross 1997; please note that she stated in 2018: “The last 20 years has not seen—as I had thought they might—a general reduction in the importance of location”, see Cairncross 2018). Viewed from another angle: entrepreneurial activities have, in the past (before the era of digitisation has begun), been considered as primarily a regional event with entrepreneurs staying in the local region where they lived and worked before starting a company—and this local region in most cases was an urban one (for good reasons as explained earlier). However, digitisation, led, at least partially, to a new situation and “digital entrepreneurship” follows very different locational logics (Nambisan et al. 2019). Digitisation unfolds countless opportunities to start a business with novel products and business models. These new companies could theoretically be founded everywhere, also in rural regions, as long as broadband Internet is available (Hasbi 2020; McCoy et al. 2018). However, there is still a significant lack of large-scale, statistically representative empirical studies on digital entrepreneurship and how it may change urban–rural disparities. On the one hand, in many countries digital infrastructure will must be significantly improved outside urban agglomerations as this is a clear goal of several governments in order to reduce spatial economic and infrastructure inequalities with all their negative social and political consequences (Rodriguez-Posé 2018). On the other hand, urban areas have partially lost in attractiveness among several young highly skilled employees due to disadvantages like high living cost, traffic, crime or low ecological quality. Supported by the recent working-from-home trend increased by the COVID-19 pandemic—an important catalysator of digitisation processes—young families in particular are more prone to live outside urban areas—as long as sufficient digital infrastructure and access to labour is provided. Combined with the obviously changed motivation structure of young entrepreneurs in high-income countries as shown in the recent GEM Global Report (see GERA 2021, between 40 and 65% of the entrepreneurs in UK, USA, Canada, Sweden, and Switzerland have started their business in order “to make a difference in the world”), there are at least some new arguments to rethink the traditional idea of entrepreneurship as a primarily urban event driven solely by economic (growth) motivations of the entrepreneurs. Thus, spatial location will remain a relevant research issue for entrepreneurship research, but under new conditions that will significantly shaped by digitisation. Applied to entrepreneurial ecosystems and following Nambisan et al. (2019), there are three key themes of digital transformation’s effect on entrepreneurship: openness, generativity, and affordance. The latter can be divided into “digital affordances” and “spatial affordances” (Autio et al. 2018), but there are only very few empirically valid proofs yet for this “novel cluster type”.

Third on the agenda, I plead for more and more explicit interdisciplinary research when it comes to geographically sensitive entrepreneurship research. As Davidsson (2016, 36) put it, “the more scholars from various disciplines invest in understanding entrepreneurship, the happier I am! […] Therefore I think we need to be a multidisciplinary community of scholars who dedicate ourselves to this phenomenon and who interact enough in order to speak roughly the same language”. I do agree! Without excluding other disciplines like psychology (see Obschonka and Stützer 2017 and Obschonka et al. 2013 for examples of a rich research stream regarding the spatiality of trait factors or the five-factor model of personality attributes, which is highly relevant for entrepreneurship activities) or sociology (see Bögenhold et al. 2014; Thornton 1999), management scholars and economists on the one hand and economic geographers on the other represent those disciplines that are primarily prone to researching the complex relationship between geography and entrepreneurship. Members of these academic disciplines, despite some encouraging examples of co-authorships, co-editorships, or cooperation in joint research projects, may in the future even more address the opportunity to work together as partners of equals, to make conciliatory moves, and to more intensively benefit from the comparative strengths of each discipline to achieve the intended synergy effects of such multidisciplinary entrepreneurship research.

From a methodological perspective, this plea for multidisciplinary entrepreneurship research is at least partially related to a plea for more multilevel entrepreneurship research (Theodoraki and Messeghem 2017). For many relevant research questions (not for all, of course), the unit of analysis should be interpreted more openly, flexibly, and additively. More often now than in the past, research designs of publications or academic projects should apply the combination of firm-level, regional-level, or industry-level attempts (hitherto dominated by economic geographers doing entrepreneurship research) on the one hand and attempts on the individual entrepreneur’s level (primarily undertaken by management scholars or economists) on the other, i.e. a multilevel perspective.

Fourth, I have assembled several rather distinct topics and methods into a rather heterogeneous group. In line with Stam (2010, 150), empirical research at the interface of geography and entrepreneurship should search for more appropriate entrepreneurship indicators because the traditional ones are “too broad, but also too narrow to capture the pursuit of entrepreneurial opportunities”. Because most empirical studies are quantitative, static, and deterministic, we also need more qualitative, dynamic, mixed methods, non-deterministic research in regional entrepreneurship. In addition, in times of globalisation and increasing streams of migrants into all regions, countries, and continents, migrant entrepreneurship will become a more relevant aspect of regional entrepreneurship than it has been in the past. Largely under-researched topics like transnational diaspora entrepreneurship stress the important bridging/broker function of entrepreneurship (Elo et al. 2018) and open up many opportunities for topics and researchers outside Anglo-America and Europe (Drori et al. 2009; Kloosterman 2010; Koh and Malecki 2016; Muñoz-Castro et al. 2019). Also, we should not overlook the many opportunities derived from the strongly increased attention government and policy-makers are paying to entrepreneurial activities. It would be helpful if empirical research on the connection between geography and entrepreneurship would more explicitly and more intensively consider the policy lessons of the main results produced before increasingly applying sophisticated entrepreneurship empirics. Do mind the transfer gap between empirical research and its transfer into entrepreneurship policy by supranational, national, regional, or local governments!

Finally (and typically for the attitude of the majority of scholars in my own discipline), it is noteworthy that according to an influential statement of (proper) economic geographers, entrepreneurship does not explicitly belong to the five themes on the economic geography research agenda that was posited some years ago in one of the most influential economic geography journals worldwide (Economic Geography 2011). While each scholar is of course free to choose the research topics he/she is focussing on in the future, this statement is not an adequate signal to the community of economic geography researchers, given the many relevant research gaps that I have identified in regional entrepreneurship.
